# Vitamin D Level and Vitamin D Receptor Genetic Variation Were Involved in the Risk of Non-Alcoholic Fatty Liver Disease: A Case-Control Study

**DOI:** 10.3389/fendo.2021.648844

**Published:** 2021-08-06

**Authors:** Ru Zhang, Minxian Wang, Min Wang, Liuxin Zhang, Yajie Ding, Zongzhe Tang, Zuqiang Fu, Haozhi Fan, Wei Zhang, Jie Wang

**Affiliations:** ^1^Department of Fundamental and Community Nursing, School of Nursing, Nanjing Medical University, Nanjing, China; ^2^Department of Nursing, The Affiliated Brain Hospital of Nanjing Medical University, Nanjing, China; ^3^Department of Epidemiology, School of Public Health, Nanjing Medical University, Nanjing, China; ^4^Department of Information, The First Affiliated Hospital of Nanjing Medical University, Nanjing, China; ^5^Department of Epidemiology, Shanghai Cancer Institute, Shanghai, China

**Keywords:** non-alcoholic fatty liver disease (NAFLD), vitamin D (VD) deficiency, 25-hydroxyvitamin D3, vitamin D receptor (VDR), genetic variation, risk assessment

## Abstract

**Background:**

It has been demonstrated that vitamin D receptor (*VDR*), a key gene in the metabolism of vitamin D (VD), may affect the development of Non-alcoholic fatty liver disease (NAFLD) by regulating VD level and its biological effects.

**Objectives:**

To investigate the effects of serum VD level, *VDR* variation, and a combination of *VDR* SNP and environmental behavior factor on the risk of NAFLD.

**Methods:**

A total of 3023 subjects from a community in Nanjing were enrolled, including 1120 NAFLD cases and 1903 controls. Serum 25(OH)D_3_ levels were measured and eight single nucleotide polymorphisms (SNPs) in *VDR* gene were genotyped.

**Results:**

Logistic regression analyses indicated that VD sufficiency and VD insufficiency were significantly associated with a low risk of NAFLD (all *P*<0.05; all *P*_trend_ *<*0.05, in a locus-dosage manner). After adjusting for gender and age, *VDR* rs2228570-A and rs11168287-A alleles were all reduced the risk of NAFLD (all *P*_FDR_=0.136, in dominant model; *P*_trend_ =0.039, combined effects in a locus-dosage manner). The protective effects of two favorable alleles were more evident among subjects ≤40 years, non-hypertension, non-hyperglycemia and non-low high density lipoprotein-cholesterol (all *P*<0.05). The area under the receiver operating curve of the combination of *VDR* SNP and exercise time for assessing NAFLD risk was slightly higher than that of only including exercise time or neither (all *P*<0.05).

**Conclusion:**

High serum VD levels and *VDR* variants (rs2228570-A and rs11168287-A) might contribute to a low risk of NAFLD in Chinese Han population. The inclusion of *VDR* SNP and exercise time could improve the efficiency in assessment of NAFLD risk, which might provide a novel perspective for early screening and preventing NAFLD.

## Introduction

Non-alcoholic fatty liver disease (NAFLD), as the most prevalent liver disease worldwide, comprises a wide disease spectrum ranging from steatosis to nonalcoholic steatohepatitis (NASH) and NASH-related cirrhosis (1, 2). The reported prevalence reaches 10-30% in the United States, Europe and Asia (3, 4). Most patients with NAFLD are asymptomatic, and only few may complain of nonspecific symptoms, like discomfort, fatigue, and vague right upper abdominal pain (5), but NAFLD can increase the risk of type 2 diabetes mellitus (T2DM) as well as cardiovascular disease (6), and NASH may develop into fibrosis, even causing cirrhosis and hepatocellular carcinoma (7). There are currently no approved therapies for NAFLD (8, 9), and it is still necessary to explore the potential influencing factors affecting the development and progression of NAFLD.

Many factors are associated with NAFLD including insulin resistance (IR), metabolic syndrome and genetic variation (10, 11). Studies have increasingly found that vitamin D (VD) can affect the development of NAFLD by regulating IR (12), immune inflammation (13), lipid metabolism and target gene expression (14). Vitamin D deficiency is implicated in NAFLD etiology (15, 16), insulin resistance (17), visceral obesity, and metabolic syndrome (18). A meta-analysis found that compared to controls, patients with NAFLD had a lower level of VD (0.36 ng/mL) and were 1.26 times more likely to be VD-deficient (19). A population-based case-control study in China found that low serum VD was associated with advanced liver fibrosis in NAFLD patients (20). However, another systematic review and meta-analysis suggested that serum VD level might not change with NAFLD histologic severity (21). A randomized, double-blind, placebo-controlled trial in Italy suggested that although high-dose oral VD supplementation could increase the serum VD level, no effect was shown on hepatic steatosis or metabolic/cardiovascular parameters in T2DM patients with NAFLD (22). Thus, the relationship between VD and NAFLD was controversial.

VD must be transported, hydroxylated and finally combined with vitamin D receptor (VDR) to accomplish its biological roles (23). Serum VD may produce different biological effects due to genetic heterogeneity, which may help to explain why some people are prone to NAFLD, while others are not (even if they have metabolic syndrome components). Thus, exploring the influence of genetic variation on NAFLD related to VD metabolism is necessary. *VDR*, a key gene in VD metabolic pathway and a member of steroid/thyroid hormone receptor family (24), transcriptionally activates target genes after binding to vitamin D responsive elements localized in the promoter regions (25). These target genes mediate various NAFLD-associated processes, including lipid and glucose metabolism, cholesterol efflux and bile acid homeostasis, hepatic fibrogenesis, cellular differentiation, apoptosis and immune response (26, 27).

Notably, NAFLD is considered a complex disease trait such that interactions between the environment and a susceptible genetic host background influence disease development and progression (2). Studying data suggests that genetic background may influence the response to lifestyle modification. For example, reduction in liver fat and liver enzyme levels in response to weight loss was larger in subjects homozygous for the PNPLA3 I148M variant compared to non-carriers (28, 29). However, there is little information available on the association between environmental behaviors and *VDR* polymorphisms in the development and progression of NAFLD. Therefore, the aim of this study was to explore the effects of serum VD level, *VDR* variation, and a combination of *VDR* SNP and environmental behavior factor on the risk of NAFLD.

## Methods

### Participants and Study Design

A total of 3023 subjects from a community in Nanjing (Jiangsu, China) were enrolled in this case-control study during July to September 2018. The NAFLD cases were recruited from those diagnosed based on “*Guideline of prevention and treatment for nonalcoholic fatty liver disease: a 2018 update*”. NAFLD can be diagnosed if the following items 1-4 coexist with the fifth or sixth item: (1) no drinking or history of overdose drinking (less than 210g/week ethanol for men and 140g/week for women in the past 12 months); (2) excluding drug hepatitis, hepatitis C virus genotype 3 infection, hepatolenticular degeneration and other specific diseases that could result in fatty liver; (3) serum levels of transaminase and γ-glutamyl transpeptidase (γ-GT) increase mildly to moderately (<5 times above the upper normal limit), usually presenting as an increase of alanine aminotransferase (ALT); (4) metabolic syndrome constituents such as visceral obesity, hyperglycemia, blood lipid disorder and hypertension; (5) the result of liver imaging study meets the imaging diagnostic criteria of diffuse fatty liver; (6) the histological findings of liver biopsy meet the pathological diagnostic criteria of fatty liver disease. The non-NAFLD controls were collected from the same community during the study period and randomly assigned to the control group. The constituent ratios of gender and age between cases and controls were considered similar, according the results of frequency-matching.

Excluded were: (1) less than 18 years old; (2) subjects with infection, acute or chronic gastrointestinal diseases, autoimmune diseases or malignant tumors; (3) subjects with history of other viral hepatitis, alcoholic liver disease or primary liver cancer; (4) subjects of excessive drinking (alcohol consumption ≥30g/d in males and ≥20g/d in females); (5) subjects who received a liver transplant within the previous year, or had complications of advanced liver disease (varicose veins, ascites, etc.); (6) subjects with drug-induced fatty hepatitis; (7) subjects with history of psychiatric disorders.

By reviewing the literature, we assumed the frequency of gene mutation in the general population was 20%, odds ratio (OR) was 1.5, two-sided test α was 0.05, and power of test (1-β) was 90%. Therefore, the minimum sample size was estimated by NCSS-PASS 11 software (Dawson edition; Kaysville, UT) to be 784. This study had a sample size large enough to guarantee the production of reliable results.

The current study protocol was in accordance with the Declaration of Helsinki, and was approved by the Institutional Ethics Review Committee of Nanjing Medical University (Nanjing, China). Written informed consent was obtained before blood test and genetic analysis.

### Collection of Basic Data and Blood Samples

Self-designed questionnaires and an electronic medical record system were used to collect the demographic and clinical characteristics of all participants. 5-mL ethylene diamine tetraacetic acid (EDTA) anticoagulant venous blood was collected from each subject in the morning after an overnight fast. Within 2 hours, the serum and blood cells in each blood sample were separated and frozen at -80°C until further serological tests and genotyping assays.

### Serum 25(OH)D_3_ Level Detection

In two groups (gender and age matched at a ratio of 1:1, 336 subjects in the NAFLD group and 336 in the control group), the serum 25(OH)D_3_ level was measured by enzyme-linked immunosorbent assay (ELISA) according to the manufacturer’s instructions (Human 25-Dihydroxy vitamin D3 (25(OH)D3) ELISA Kit; Jin Yibai Biological Technology Co., Ltd.; Nanjing, China).

### SNP Selection and Genotyping

Target SNPs with potential biological function were selected to better identify the susceptibility sites that affect the development of NAFLD. The selection processes were as follows: (1) Downloading the genotype database of *VDR* gene in Han Chinese in Beijing (CHB) from the 1000 Genomes Project database (http://asia.ensembl.org/Homo_sapiens/Info/Index); (2) Importing the genotype database into the Haploview software (version 4.2; Broad Institute, Cambridge, MA, USA). Setting parameters: Hardy-Weinberg *P*-value cutoff = 0.05; minor allele frequency (MAF) = 0.05; r^2^ threshold = 0.8. At this point, 56 tagging SNPs (tagSNPs) were captured; (3) Searching for literature in which these potential tagSNPs were associated with NAFLD, hypertension, type 2 diabetes, hyperlipidemia, metabolic syndrome, inflammatory diseases or immune-related disorders. Finally, eight disease-related target SNPs of *VDR* gene were selected, including rs3782905 (C>G), rs3847987 (C>A), rs11574129 (T>C), rs2228570 (C>A), rs11568820 (G>A), rs739837 (G>T), rs7975232 (C>A) and rs11168287 (G>A).

Genomic DNA was isolated from EDTA anticoagulated blood samples using magnetic bead method (blood genomic extraction kit; Pangu Genome Nanotechnology Co., Ltd.; Nanjing, China). The eight SNPs were genotyped using TaqMan allelic discrimination assays on the ABI 9700 system (Applied Biosystems, Foster City, California, USA; catalog numbers: C:_3290647_10, C:_2404006_10, C_175992105_10, C:12060045_20, C:_2880808_10, C:_2404007_10, C:28977635_10, C:_2404006_10). The quality control of experimental data was as follows: (1) blinding was adopted in genotyping, so that all laboratory personnel were unclear about the clinical data of the subjects; (2) 10% of the samples were randomly selected for repeated experiments with a repeatability of 100%. The success rates of genotyping all SNPs were higher than 99% in this study.

### Statistical Analysis

All statistical analyses were processed using SPSS (version 22.0, SPSS Inc., Chicago, IL, USA) and MedCalc (Version 19.1, Ostend, Belgium). Distributions of demographic and clinical characteristics of two groups were compared using *χ* ^2^-test, student’s *t* test or Mann-Whitney U test, wherever appropriate. Logistic regression analysis was used to calculate odds ratio (OR) and 95% confidence interval (95% CI) for quantifying the association of serum 25(OH)D_3_ level with the risk of NAFLD. The correlation of serum 25(OH)D_3_ level with *VDR* SNPs was assessed using general linear regression model adjusted for gender and age. The Hardy-Weinberg equilibrium (HWE) was tested using a goodness of-fit *χ* ^2^ -test among the control subjects. The relationships between *VDR* SNPs and the risk of NAFLD were analyzed by dominant model (heterozygote + mutant homozygote *VS*. wild homozygote), recessive model (mutant homozygote *VS*. wild homozygote + heterozygote), and additive model (mutant homozygote *VS*. heterozygote *VS*. wild homozygote), respectively. For multiple SNPs comparisons, false discovery rate (FDR) correction was used and the *P*_FDR_ value ≤ 0.25 was regarded as modest confidence that the correlation represented a positive result (30). Subgroup analysis was performed for positive SNPs, and Q test was performed to calculate the heterogeneity between subgroups. The area under the receiver operating curves (AUROCs) was performed to assess the predictive power of the combination of positive SNPs, environmental behavior factors and mainly related clinical factors for NAFLD risk. A two-tailed test with a *P* value < 0.05 was regarded as statistically significant in all analyses.

## Results

### Basic Characteristics of Participants

The demographic and clinical characteristics of 1120 NAFLD cases and 1903 controls were summarized in **Table 1**. No significant differences were observed in the distribution of gender and age between the two groups (all *P*>0.05). However, there were significant differences in exercise time, body mass index (BMI), waist circumference (WC), systolic blood pressure (SBP), diastolic blood pressure (DBP), triglyceride (TG), total cholesterol (TC), high density lipoprotein-cholesterol (HDL-C), low density lipoprotein-cholesterol (LDL-C), glucose (GLU), γ-glutamyl transpeptidase (γ-GT), alanine aminotransferase (ALT), aspartate aminotransferase (AST), direct bilirubin (DBIL), total bilirubin (TBIL) and serum 25(OH)D_3_ levels (all *P*<0.05).

**Table 1:** Distributions and comparisons of demographic and clinical characteristics between NAFLD case and control groups.

Variables	NAFLD cases (N=1120)	Controls (N=1903)	*χ^2^*/*t*/*Z*	*P*
Gender			2.324	0.127^a^
male	948 (84.6)	1570 (82.5)		
female	172 (15.4)	333 (17.5)		
Age (years)			1.107	0.293^a^
≤40	596 (53.2)	975 (51.2)		
>40	524 (46.8)	928 (48.8)		
mean ± SD	40.48 ± 8.80	39.89 ± 9.78	-1.649	0.099^b^
Exercise time (min/week)			**8.556**	**0.003** ^a^
<150	622 (58.6)	995 (53.0)		
≥150	440 (41.4)	883 (47.0)		
mean ± SD	144.09 ± 41.77	150.06 ± 43.82	**3.609**	**<0.001** ^b^
BMI (kg/m^2^)	25.36 ± 2.53	22.80 ± 2.48	**-27.235**	**<0.001** ^b^
WC (cm)	88.47 ± 7.85	81.68 ± 7.59	**-23.462**	**<0.001** ^b^
SBP (mmHg)	130.58 ± 15.08	124.70 ± 13.70	**-10.700**	**<0.001** ^b^
DBP (mmHg)	79.48 ± 10.86	74.81 ± 9.38	**-12.134**	**<0.001** ^b^
TG (mmol/L)	1.96 (1.43, 2.69)	1.06 (0.79, 1.41)	**-23.408**	**<0.001** ^c^
TC (mmol/L)	4.84 (4.27, 5.42)	4.45 (4.05, 5.03)	**-7.804**	**<0.001** ^c^
HDL-C (mmol/L)	1.00 (0.85, 1.18)	1.27 (1.07, 1.48)	**-18.179**	**<0.001** ^c^
LDL-C (mmol/L)	2.82 (2.20, 3.33)	2.63 (2.24, 3.10)	**-4.424**	**<0.001** ^c^
GLU (mmol/L)	3.13 (2.39, 5.00)	3.91 (2.32, 4.73)	**-5.754**	**<0.001** ^c^
γ-GT (U/L)	35.00 (24.00, 55.00)	17.00 (13.00, 25.00)	**-20.121**	**<0.001** ^c^
ALT (U/L)	36.00 (25.00, 54.50)	18.00 (13.25, 25.00)	**-19.872**	**<0.001** ^c^
AST (U/L)	24.00 (19.00, 31.00)	19.00 (16.00, 21.00)	**-11.789**	**<0.001** ^c^
DBIL (µmol/L)	4.01 (3.21 5.21)	4.20 (3.32, 5.23)	**-5.025**	**<0.001** ^c^
TBIL (µmol/L)	13.59 (10.71, 18.39)	13.85 (11.21, 18.21)	**-3.267**	**0.001** ^c^
25(OH)D_3_ (ng/mL)^d^			**22.575**	**<0.001** ^a^
<20	130 (71.0)	167 (49.7)		
20-30	27 (14.8)	97 (28.9)		
≥30	26 (14.2)	72 (21.4)		
median (IQR)	16.06 (12.21, 20.78)	20.00 (16.84, 26.68)	**-7.168**	**<0.001** ^c^

NAFLD, non-alcoholic fatty liver disease; SD, standard deviation; BMI, body mass index; WC, waist circumference; SBP, Systolic blood pressure; DBP, Diastolic blood pressure; TG, triglyceride; TC, total cholesterol; HDL-C, high density liptein cholesterol; LDL-C, low density lipoprotein-chesterol; GLU, glucose; γ-GT, γ-glutamyl transpeptidase; ALT, alanine aminotransferase; AST, aspartate aminotransferase; DBIL, direct bilirubin; TBIL, total bilirubin; 25(OH)D_3_, 25-hydroxyvitamin D3; IQR, interquartile range.

a χ^2^-test among two groups.

b Student’s t test among two groups.

c Mann-Whitney U test among two groups.

d serum 25(OH)D_3_ levels were measured in 336 NAFLD cases and 336 controls.

Bold type indicates statistically significant results.

### Association Between Serum 25(OH)D_3_ Level and NAFLD Risk

According to the level of serum 25(OH)D_3_, 672 participants were divided into three categories: VD deficiency (<20ng/mL), VD insufficiency (20ng/mL-30ng/mL) and VD sufficiency (≥30ng/mL) (31). The constituent ratios of three categories were 60.9% (409/672), 21.4% (144/672), and 17.7% (119/672), respectively. The prevalences of NAFLD in the three categories were 59.2% (242/409), 32.6% (47/144) and 39.5% (47/199), respectively, with significant differences (*χ* ^2^ = 36.366, *P*<0.05). In addition, multiple comparisons showed that the prevalence of NAFLD in patients with VD deficiency was significantly higher than that in patients with VD insufficient or VD sufficient (all *P*_Bonferroni_<0.017), but there was no significant difference in the prevalence of NAFLD between categories with VD insufficiency and VD sufficiency (*P*_Bonferroni_>0.017) (**Supplementary Figure 1**).

Further logistic regression analysis suggested that compared to VD deficiency, VD sufficiency (crude OR=0.450, 95%CI=0.297-0.684, *P*<0.001) and VD insufficiency (crude OR=0.334, 95%CI=0.224-0.499, *P*<0.001) were associated with a lower risk of NAFLD, and this association was dose-dependent (*P*_trend_<0.001) (model 1). After adjusting for gender, age, BMI and SBP (model 2), the subjects with VD sufficiency (adjusted OR=0.477, 95%CI=0.277-0.821, *P*=0.008) and VD insufficiency (adjusted OR=0.344, 95%CI=0.206-0.573, *P*<0.001) all had significantly decreased risk of NAFLD compared with those with VD deficiency, and a significant locus-dosage effect of VD level on NAFLD risk was also observed (*P*_trend_<0.001). When adjusting for the above factors and TG, HDL-C, GLU (model 3), the risk of NAFLD still decreased significantly with the increase of VD level (VD sufficiency vs. VD deficiency: adjusted OR=0.552, 95%CI=0.305-0.998, *P*=0.049; VD insufficiency vs. VD deficiency: adjusted OR=0.298, 95%CI=0.165-0.539, *P*<0.001; *P*_trend_=0.005) (**Table 2**).

**Table 2:** Logistic regression analysis of the associations between serum 25(OH)D_3_ levels and risk of NAFLD.

	serum 25 (OH)D_3_ levels			*P*_trend_
	VD deficiency (N=409)	VD insufficiency (N=144)	VD sufficiency (N=119)
Model 1	1.00 (Ref)	**0.334 (0.224, 0.499)**	**0.450 (0.297, 0.684)**	**<0.001**
*P*		**<0.001**	**<0.001**	
Model 2	1.00 (Ref)	**0.344 (0.206, 0.573)**	**0.477 (0.277, 0.821)**	**<0.001**
*P*		**<0.001**	**0.008**	
Model 3	1.00 (Ref)	**0.298 (0.165, 0.539)**	**0.552 (0.305, 0.998)**	**0.005**
*P*		**<0.001**	**0.049**	

NAFLD, non-alcoholic fatty liver disease; 25(OH)D_3_, 25-hydroxyvitamin D3.

VD deficiency, serum 25(OH)D_3_ < 20ng/mL; VD insufficiency, 20ng/mL≤ serum 25(OH)D_3_ <30ng/mL; VD sufficiency, serum 25(OH)D_3_ ≥30ng/mL.

Model 1, unadjusted; Model 2, adjusted for gender, age, BMI, SBP; Model 3, model 2 with additional adjustment for TG, HDL-C, GLU.

Bold type indicates statistically significant results.

### Associations Between *VDR* SNPs and Serum 25(OH)D_3_ Level

Serum 25(OH)D_3_ levels were Lg transformed into an approximately normal distribution, and SNPs were coded in an additive genetic model. The general linear regression analysis indicated that there were no significant associations between eight *VDR* SNPs and serum 25(OH)D_3_ levels after adjusting for gender and age (all *P*>0.05) (**Supplementary Table 1**).

### Association Between *VDR* SNPs and NAFLD Risk

The genotype distributions of the eight SNPs in both groups were shown in **Table 3**. After adjusting for gender and age, logistic regression analyses showed that *VDR* rs2228570-A variant (AA: adjusted OR=0.782, 95%CI=0.633-0.966, *P*=0.023; dominant model: adjusted OR=0.837, 95%CI=0.710-0.986, *P*=0.034; additive model: adjusted OR=0.883, 95%CI=0.794-0.981, *P*=0.020) and rs11168287-A variant (GA: adjusted OR=0.830, 95%CI=0.707-0.974, *P*=0.022; dominant model: adjusted OR=0.839, 95%CI=0.721-0.976, *P*=0.023) all significantly reduced the risk of NAFLD in different models. Under the premise of FDR threshold ≤ 0.25, FDR revealed with modest confidence that the associations between the two SNPs and a low risk of NAFLD were positive (all *P*_FDR_=0.136, in dominant model; **Supplementary Table 2**).

**Table 3:** Genotype distributions of *VDR* polymorphisms among the two study groups and association analyses of these eight SNPs and NAFLD.

SNP	NAFLD cases n (%)	Controls n (%)	OR (95% CI)^a^	*P* ^a^
rs3782905-CC	756 (69.3)	1328 (71.1)	1.00 (Ref)	
CG	296 (27.1)	475 (25.4)	1.087 (0.922, 1.282)	0.319
GG	39 (3.6)	65 (3.5)	0.980 (0.773, 1.244)	0.870
Dominant model			1.061 (0.909, 1.238)	0.455
Recessive model			0.940 (0.752, 1.175)	0.586
Additive model			1.015 (0.909, 1.133)	0.794
rs3847987-CC	673 (61.5)	1172 (62.7)	1.00 (Ref)	
CA	372 (34.0)	619 (33.1)	1.065 (0.907, 1.252)	0.442
AA	49 (4.5)	79 (4.2)	1.060 (0.728, 1.543)	0.761
Dominant model			1.065 (0.912, 1.244)	0.428
Recessive model			1.037 (0.715,1.503)	0.848
Additive model			1.050 (0.921, 1.197)	0.462
rs11574129-TT	752 (68.6)	1266 (67.2)	1.00 (Ref)	
TC	311 (28.4)	569 (30.2)	0.924 (0.783, 1.090)	0.347
CC	34 (3.1)	48 (2.5)	1.212 (0.777, 1.893)	0.397
Dominant model			0.946 (0.806, 1.111)	0.501
Recessive model			1.241 (0.797, 1.933)	0.338
Additive model			0.979 (0.851, 1.127)	0.769
rs2228570-CC	339 (30.6)	518 (27.5)	1.00 (Ref)	
CA	537 (48.8)	933 (49.6)	0.863 (0.725, 1.028)	0.099
AA	225 (20.4)	431 (22.9)	**0.782 (0.633, 0.966)**	**0.023**
Dominant model			**0.837 (0.710, 0.986)**	**0.034**
Recessive model			0.857 (0.715, 1.028)	0.097
Additive model			**0.883 (0.794, 0.981)**	**0.020**
rs11568820-GG	372 (33.9)	596 (31.7)	1.00 (Ref)	
GA	550 (50.1)	951 (50.6)	0.947 (0.801, 1.118)	0.519
AA	176 (16.0)	331 (17.6)	0.833 (0.664, 1.044)	0.113
Dominant model			0.917 (0.783, 1.075)	0.285
Recessive model			0.861 (0.703, 1.054)	0.148
Additive model			0.919 (0.824, 1.025)	0.128
rs739837-GG	566 (51.7)	983 (52.3)	1.00 (Ref)	
GT	448 (40.9)	767 (40.8)	1.045 (0.893, 1.224)	0.583
TT	81 (7.4)	131 (7.0)	1.097 (0.817, 1.472)	0.540
Dominant model			1.053 (0.906, 1.224)	0.500
Recessive model			1.076 (0.808, 1.433)	0.616
Additive model			1.046 (0.929, 1.179)	0.457
rs7975232-CC	564 (51.4)	978 (51.9)	1.00 (Ref)	
CA	446 (40.6)	770 (40.9)	1.003 (0.857, 1.175)	0.967
AA	88 (8.0)	135 (7.2)	1.118 (0.848, 1.474)	0.428
Dominant model			1.023 (0.880, 1.188)	0.770
Recessive model			1.116 (0.854, 1.459)	0.420
Additive model			1.035 (0.921, 1.162)	0.566
rs11168287-GG	466 (42.6)	730 (38.9)	1.00 (Ref)	
GA	486 (44.5)	903 (48.1)	**0.830 (0.707, 0.974)**	**0.022**
AA	141 (12.9)	243 (13.0)	0.872 (0.689, 1.104)	0.255
Dominant model			**0.839 (0.721, 0.976)**	**0.023**
Recessive model			0.963 (0.773, 1.200)	0.738
Additive model			0.904 (0.810, 1.009)	0.071

VDR, vitamin D receptor; SNPs, single nucleotide polymorphisms; NAFLD, non-alcoholic fatty liver disease; OR, odds ratio; CI, confidence interval.

A pair of alleles such as C/G, if G is a less frequent gene, then dominant model (CG+GG vs. CC), recessive model (GG vs. CG+CC), and additive model (GG vs CG vs. CC).

a Logistic regression model, adjusted for gender and age.

Bold type indicates statistically significant results.

In addition, the combined effects of *VDR* variants rs2228570 and rs11168287 on the risk of NAFLD were estimated by the number of favorable alleles from the two SNPs, as shown in **Supplementary Table 3**. The subjects were first divided into three groups with “0”, “1-2” and “3-4” favorable alleles. Compared with those who had 0 favorable alleles, subjects with “3-4” favorable alleles had significantly decreased risk of NAFLD (adjusted OR=0.750, 95%CI=0.577-0.974, *P*=0.031) and the more favorable alleles, the lower NAFLD risk, suggesting a significant locus-dosage effect of the combined alleles on NAFLD risk (*P*_trend_=0.039). The subjects were then distributed into two groups with “0” and “1-4” favorable alleles, and we found that the presence of “1-4” alleles was related to a 0.798-fold lower risk of NAFLD (adjusted 95% CI=0.644-0.990, *P*=0.040).

We further performed the stratification analyses to evaluate the combined effects of *VDR* rs2228570-A and rs11168287-A alleles on the risk of NAFLD adjusted with gender and age. As shown in **Supplementary Figure 2**, the combined protective effects of two alleles were more prominent in subjects ≤ 40 years (adjusted OR=0.696, 95%CI=0.515-0.940, *P*=0.018), non-hypertension (adjusted OR=0.770, 95%CI=0.602-0.986, *P*=0.038), non-hyperglycemia (adjusted OR=0.791, 95%CI=0.631-0.991, *P*=0.042) and non-low HDL-C (adjusted OR=0.710, 95%CI=0.536-0.942, *P*=0.018). The heterogeneity test discovered no significant differences among all the subgroups (all *P*>0.05).

### Influence Factors of NAFLD

A stepwise regression model were performed with gender, age, visceral obesity, hypertension, hyperglycemia, hypertriglyceridemia, Low HDL-C, ALT, exercise time, rs2228570 and rs11168287. The coding of each variable was described in **Supplementary Table 4**. The results showed that age ≤40 years (OR=0.716, 95%CI=0.583-0.879, *P*=0.001), exercise time ≥150 min/week (OR=0.798, 95%CI=0.661-0.963, *P*=0.019) and rs2228570-A (OR=0.768, 95%CI=0.626-0.942, *P*=0.011) were independent protective factors of NAFLD. Conversely, visceral obesity (OR=3.653, 95%CI=2.984-4.471, *P*<0.001), hypertension (OR=1.654, 95%CI=1.290-2.121, *P*<0.001), hypertriglyceridemia (OR=3.455, 95%CI=2.784-4.288, *P*<0.001), Low HDL-C (OR=1.879, 95%CI=1.536-2.299, *P*<0.001) and ALT > 40U/L (OR=2.729, 95%CI=2.060-3.615, *P*<0.001) were independent risk factors of NAFLD (**Table 4**).

**Table 4:** Multivariate stepwise regression analysis for independent factors of NAFLD.

Variables	*β*	SE	Wald	OR (95% CI)	*P*
Age (≤40 *vs.* >40 years)	-0.334	0.105	10.155	**0.716 (0.583, 0.879)**	**0.001**
Visceral obesity	1.296	0.103	157.695	**3.653 (2.984, 4.471)**	**<0.001**
Hypertension	0.503	1.27	15.733	**1.654 (1.290, 2.121)**	**<0.001**
Hypertriglyceridemia	1.240	0.110	126.586	**3.455 (2.784, 4.288)**	**<0.001**
Low HDL-C	0.631	0.103	37.538	**1.879 (1.536, 2.299)**	**<0.001**
ALT (>40 *vs.*≤40U/L)	1.004	0.143	48.990	**2.729 (2.060, 3.615)**	**<0.001**
Exercise time (≥150 *vs.* <150 min/week)	-0.225	0.096	5.511	**0.798 (0.661, 0.963)**	**0.019**
rs2228570 (CA+AA *vs.*CC)	-0.264	0.104	6.422	**0.768 (0.626, 0.942)**	**0.011**

NAFLD, non-alcoholic fatty liver disease; HDL-C, high density liptein cholesterol; ALT, alanine aminotransferase; OR, odds ratio; CI, confidence interval.

Bold type indicates statistically significant results.

**Figure 1:**
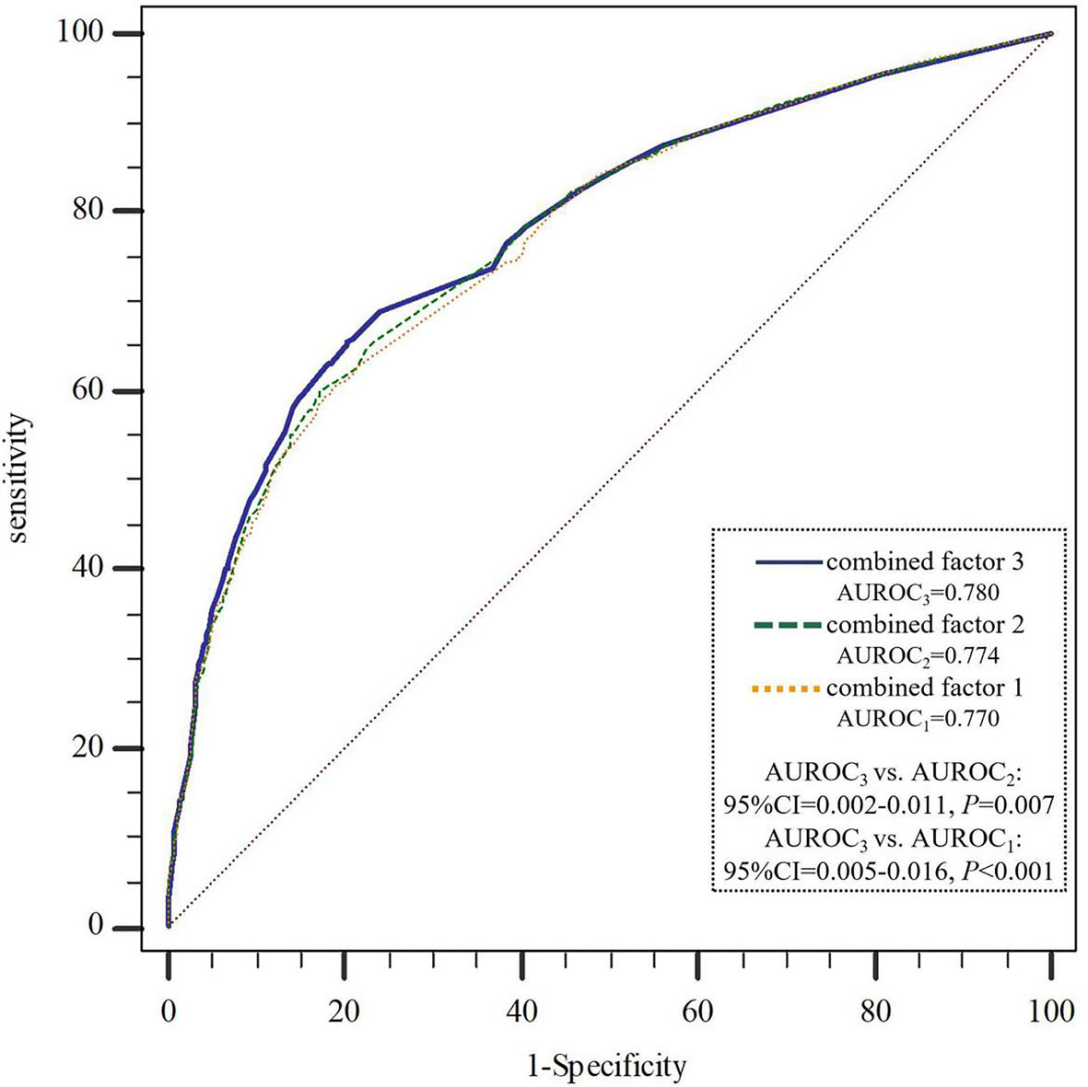
AUROCs comparison of the three combined factors to assess NAFLD risk. NAFLD, non-alcoholic fatty liver disease; AUROC, area under the receiver operating curve. combined factor 1: a combination of age, visceral obesity, blood pressure, TG, HDL-C and ALT; combined factor 2: a combination of combined factor 1 and exercise time; combined factor 3: a combination of combined factor 2 and *VDR*-rs2228570.

We further constructed combined factors based on the above factors for assessing NAFLD risk, and the combined factor 1 (a combination of clinical factors) = (-1.588) + (-0.229) × age + 1.333 × visceral obesity + 0.484 × hypertension + 1.233 × hypertriglyceridemia + 0.621 × Low HDL-C + 0.939 × ALT. Similarly, combined factor 2 (a combination of clinical factors and exercise time) = (-1.448) + (-0.254) × age + 1.294 × visceral obesity + 0.512 × hypertension + 1.222 × hypertriglyceridemia + 0.610 × low HDL-C + 0.958 × ALT + (-0.220) × exercise time; combined factor 3 (a combination of clinical factors, exercise time and SNP genetic variant) = (-1.519) + (-0.334) × age + 1.296 × visceral obesity + 0.503 × hypertension + 1.240 × hypertriglyceridemia + 0.631 × low HDL-C + 1.004 × ALT + (-0.225) × exercise time + (-0.264) × rs2228570. In **Figure 1**, the AUROCs of these combined factors were 0.770, 0.774, and 0.780, respectively, and the AUROC of combined factor 3 for assessing NAFLD risk was slightly higher than that of combined factor 2 (0.780 vs. 0.774, 95%CI=0.002-0.011, *P*=0.007) and combined factor 1 (0.780 vs. 0.770, 95%CI=0.005-0.016, *P*<0.001).

## Discussion

In the current study, our findings suggested that high serum vitamin D level, *VDR* rs2228570-A and rs11168287-A were associated with a decreased risk of NAFLD, and the combination of *VDR* rs2228570 and exercise time was more effective in the risk assessment of NAFLD.

Accumulative evidence suggests that VD deficiency is highly prevalent among the general population in China (32), and low VD level is a risk factor of NAFLD (33). VD poses protective effects on many other metabolic-related diseases, such as obesity, hypertension, insulin resistance, type 2 diabetes, metabolic syndrome and cardiovascular disease (34–36). Consistently, our study manifested a high prevalence of NAFLD (59.2%) in the subjects with low serum 25(OH)D_3_ level (<20ng/mL), and VD deficiency might increase the risk of NAFLD. The regulatory mechanism of VD level and *VDR* activity in the development of NAFDL has not been fully elucidated yet (37, 38). Therefore, the present study provides a new insight into the association between *VD*R genetic mutation and NAFLD risk.

The *VDR* SNPs rs7975232, rs2228570 were reported to be associated with VD deficiency (39, 40). However, other research showed no association of rs7975232, rs11568820, rs11574129 with serum 25(OH)D_3_ in a Han Chinese population (41). A case-control study in Caucasian population found no correlation between VD level and rs2228570 genotype (42). In our study, no significant associations were found between eight *VDR* polymorphisms and 25(OH)D_3_ level either. Possible explanations include geographical location, racial backgrounds and sunlight exposure (43).

Our study suggested that the SNPs of rs2228570-A and rs11168287-A in *VDR* gene exhibited an association with the decrease in NAFLD risk, with an evident gene-gene combined effect. Metabolic impairment and alteration of the glucose-insulinhomeostasis are the primarily pathogenesis of NAFLD (44). Evidence indicated that the rs2228570 polymorphism of *VDR* gene was associated with the risk of fasting glucose in a Chinese Han population (45). It was found that the expression of *VDR* mRNA in the liver of obese individuals with biopsy-proven NAFLD was higher than that of non-NAFLD (46). Study reported that the combination of rs2228570 (*FokI*), rs1544410 (*BsmI*) and rs731236 (*TaqI*) was involved in the risk of T2DM in north Indians (47). However, another study showed that *VDR*-rs2225780 genetic variation was not associated with T2DM in a Caucasian population (42). The possible explanation might have various ethnic groups. The Jackson Heart Study indicated that *VDR* variants are associated with abdominal visceral adipose tissue volume and adiponectin concentrations, but not with BMI or WC in African Americans (48), a finding that is consistent with the stratified analysis of the combined effect of the two positive SNPs in this study. Although there is no research on rs11168287 polymorphisms and NAFLD, we found that rs11168287 is situated in the peak of H3k4me1 and near the peak of transcription levels by searching the UCSC genome browser (https://genome.ucsc.edu), which means that rs11168287 mutation might affect the serum VD level and its biological effects including insulin sensitivity, lipid metabolism as well as immune inflammation by dysregulating the transcription and expression of *VDR* gene, and ultimately affect the risk of NAFLD. Further study is warranted to identify the role and function of these *VDR* polymorphisms in VD metabolism and NAFLD risk.

This study also demonstrated that age ≤40 years, exercise time ≥150 min/week, and rs2228570-A were independent protective factors of NAFLD; visceral obesity, hypertension, hypertriglyceridemia, Low HDL-C and ALT >40U/L were independent risk factors of NAFLD, which is consistent with the results of previous studies (49–52). Based on these independent factors, we constructed combined factors to assess NAFLD risk. The results showed that the assessment efficiency of combined factor 3 (including age, visceral obesity, blood pressure, TG, HDL-C, ALT, exercise time and rs2228570) in NAFLD risk was higher than that of combined factor 2 (including age, visceral obesity, blood pressure, TG, HDL-C, ALT and exercise time) and combined factor 1 (including age, visceral obesity, blood pressure, TG, HDL-C and ALT). There is no research on the combination of genetic and environmental behavioral factors to evaluate the risk of NAFLD in Chinese population so far. A study of NAFLD risk in Italian obese children and adolescents found that combining genetic variants with clinical risk factors improved the predictability of NAFLD (53). For a disease related to heredity and environment, the predictive power of genetic-environmental factors is reasonably stronger than a single one. However, in our study, compared with the AUROC of combined factor 1 or combined factor 2, the AUROC of combined factor 3 only increased slightly, probably due to the weak effect of a single SNP on disease risk (54). In spite of this, we cannot ignore the effect of individual differences in genetic mutation on disease susceptibility. Once the risk of a particular genotype and environmental exposure combination is known, medical interventions, including medical surveillance, lifestyle advice, diet or drug treatment, could then be taken for high-risk groups or individuals to prevent disease (55, 56).

There are a few limitations in our study. Firstly, based on the case-control study, we collected the data of the subjects and measured their VD level, which may not represent the average level of different seasons. A convenient and inexpensive ELISA method was used to detect 25(OH)D_3_ levels in this study. Although it is not as accurate as liquid chromatography tandem mass spectrometry (LC-MS), it can reflect the level of 25(OH)D_3_ to some extent (41, 57). Secondly, only subjects from one community were enrolled, which may not be representative enough. In response to this problem, the frequency-matching of gender and age was used in the design stage, and multivariate analysis and stratified analysis were carried out to control the influence of the confounding factors to a certain extent. Finally, we only selected one key gene in the VD metabolic pathway, which may not be able to fully analyze the relationship between genetic factors and NAFLD risk. It is necessary to further explore the impact of polygenic polyloci and their combination with environmental factors on disease risk in a multicenter population of different races.

In conclusion, our results supported that high serum VD levels and *VDR* variants (rs2228570-A and rs11168287-A) might be involved in a low risk of NAFLD in the Chinese Han population, and a combination of *VDR* SNP and exercise time could improve the efficiency in assessment of NAFLD risk. These findings might provide new insight for risk evaluation of NAFLD and early screening of high-risk population.

## Data Availability Statement

The original contributions presented in the study are included in the article/**Supplementary Material**. Further inquiries can be directed to the corresponding author.

## Ethics Statement

The studies involving human participants were reviewed and approved by The Institutional Ethics Review Committee of Nanjing Medical University (Nanjing, China). The patients/participants provided their written informed consent to participate in this study.

## Author Contributions 

JW designed and organized the study. RZ, MXW, MW, LZ, YD, ZT, ZF, HF, and WZ contributed to the planning, designing and analyses of the experiments, data collection and quality control. RZ, MXW, and JW wrote and critical revised the manuscript. All authors contributed to the article and approved the submitted version.

## Funding

This work was supported by the Natural Science Foundation of Jiangsu Province, China (Grant No. BK20181369) and Priority Academic Program Development of Juangsu Higher Education Institutions (Grant No. PAPD [2018] 87).

## Conflict of Interest

The authors declare that the research was conducted in the absence of any commercial or financial relationships that could be construed as a potential conflict of interest.

## Publisher’s Note

All claims expressed in this article are solely those of the authors and do not necessarily represent those of their affiliated organizations, or those of the publisher, the editors and the reviewers. Any product that may be evaluated in this article, or claim that may be made by its manufacturer, is not guaranteed or endorsed by the publisher.
