# Supplementary material for: Vitamin D Level and Vitamin D Receptor Genetic Variation Were Involved in the Risk of Non-Alcoholic Fatty Liver Disease: A Case-Control Study
Source: Front Endocrinol (Lausanne). 2021 Aug 6;12:648844. doi: 10.3389/fendo.2021.648844 (PMC8377425; doi:10.3389/fendo.2021.648844)
Supplement: Supplementary file 1 [file DataSheet_1.doc]

| **Supplementary Table 1** Associations of serum 25(OH)D_3_ levels with *VDR* SNPs | | | |
| --- | --- | --- | --- |
| SNPs | Allele | Beta coefficient (95% CI)^a^ | *P*^a^ |
| rs3782905 | C/G | +0.011 (-0.016, 0.038) | 0.417 |
| rs3847987 | C/A | -0.025 (-0.056, 0.006) | 0.112 |
| rs11574129 | T/C | -0.024 (-0.057, 0.009) | 0.148 |
| rs2228570 | C/A | +0.018 (-0.008, 0.044) | 0.183 |
| rs11568820 | G/A | -0.014 (-0.041, 0.013) | 0.297 |
| rs739837 | G/T | -0.010 (-0.039,0.018) | 0.472 |
| rs7975232 | C/A | -0.013 (-0.041, 0.015) | 0.351 |
| rs11168287 | G/A | -0.012 (-0.038, 0.014) | 0.376 |

**Notes.**

*Abbreviations:* 25(OH)D_3_, 25-hydroxyvitamin D3; *VDR*, vitamin D receptor; SNPs, single nucleotide polymorphisms; CI, confidence interval.

Serum 25(OH)D_3_ levels were Lg transformed to be an approximately normal distribution.

Allele: major allele/minor allele.

^a^ General linear regression, adjusted for gender and age.

| **Supplementary Table 2** Associations of *VDR* SNPs with NAFLD risk in different models of multivariable analyses | | | | | | |
| --- | --- | --- | --- | --- | --- | --- |
| SNPs | Dominant model | | Recessive model | | Additive model | |
|  | *P* | *P*_FDR_^a^ | *P* | *P*_FDR_^a^ | *P* | *P*_FDR_^a^ |
| rs3782905 | 0.455 | 0.573 | 0.586 | 0.821 | 0.794 | 0.794 |
| rs3847987 | 0.428 | 0.573 | 0.848 | 0.848 | 0.462 | 0.739 |
| rs11574129 | 0.501 | 0.573 | 0.338 | 0.821 | 0.769 | 0.794 |
| rs2228570 | **0.034** | **0.136** | 0.097 | 0.592 | **0.020** | **0.160** |
| rs11568820 | 0.285 | 0.573 | 0.148 | 0.592 | 0.128 | 0.341 |
| rs739837 | 0.500 | 0.573 | 0.616 | 0.821 | 0.457 | 0.739 |
| rs7975232 | 0.770 | 0.770 | 0.420 | 0.821 | 0.566 | 0.755 |
| rs11168287 | **0.023** | **0.136** | 0.738 | 0.843 | 0.071 | 0.284 |

**Notes.**

*Abbreviations*: *VDR*, vitamin D receptor; SNPs, single nucleotide polymorphisms; NAFLD, non-alcoholic fatty liver disease; FDR, false discovery rate.

^a^ FDR was used and the *P*-value was adjusted to 0.25.

Bold type indicates statistically significant results, deriving from logistic regression analyses with adjustment for gender and age.

| **Supplementary Table 3** Combined effects of rs2228570 and rs11168287 on risk of NAFLD | | | | |
| --- | --- | --- | --- | --- |
| Favorable allele number ^a^ | NAFLD cases  n (%) | Controls  n (%) | OR (95%CI)^b^ | *P*^b^ |
| 0 | 166 (14.8) | 232 (12.2) | 1.00 (Ref) |  |
| 1-2 | 749 (66.9) | 1288 (67.7) | 0.813 (0.653, 1.012) | 0.063 |
| 3-4 | 205 (18.3) | 382 (20.1) | **0.750 (0.577, 0.974)** | **0.031** |
| Trend |  |  |  | *P*_trend_=**0.039**^c^ |
| 0 | 166 (14.8) | 232 (12.2) | 1.00 (Ref) |  |
| 1-4 | 954 (85.2) | 1670 (87.8) | **0.798 (0.644, 0.990)** | **0.040** |

**Notes.**

*Abbreviations:* NAFLD, non-alcoholic fatty liver disease; OR, odds ratio; CI, confidence interval.

^a^ The favorable alleles: rs2228570-A and rs11168287-A.

^b^ Logistic regression analysis was adjusted for gender and age.

^c^ *P*-value of Cochran-Armitahge’s trend test.

Bold type indicates statistically significant results.

| **Supplementary Table 4** The coding of variables for NAFLD predictors | |
| --- | --- |
| Predictor variables | Variable assignments |
| gender | Male=0, Female=1 |
| age | ≤40 years=0, >40 years=1 |
| visceral obesity | No =0, Yes=1 |
| hypertension | No =0, Yes=1 |
| hyperglycemia | No =0, Yes=1 |
| hypertriglyceridemia | No =0, Yes=1 |
| Low HDL-C | No =0, Yes=1 |
| exercise time | <150min/week=0, ≥150 min/week=1 |
| ALT | ≤40U/L=0, >40U/L=1 |
| rs2228570 | CC=0, CA+AA=1 |
| rs11168287 | GG=0, GA+AA=1 |

**Notes.**

*Abbreviations:* NAFLD, non-alcoholic fatty liver disease; HDL-C, high density liptein cholesterol; ALT, alanine aminotransferase.


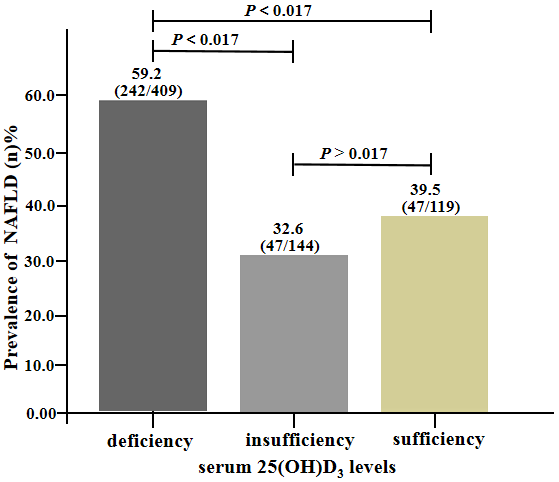


**Supplementary Figure1.** **Prevalence of NAFLD according to serum 25(OH)D_3_ level.**

*Abbreviations:* NAFLD, non-alcoholic fatty liver disease; VD, vitamin D; 25(OH)D_3_,

25-hydroxyvitamin D3.

VD deficiency, serum 25(OH)D_3_ < 20ng/mL; VD insufficiency, 20ng/mL≤ serum 25(OH)D_3_ <30ng/mL; VD sufficiency, serum 25(OH)D_3_ ≥30ng/mL.

*χ^2^*-test among three groups, *χ^2^* = 36.366, *P*<0.05.

Z-test between any two groups, bonferroni correction was applied and the *P*-value was adjusted to 0.017 (0.05/3).


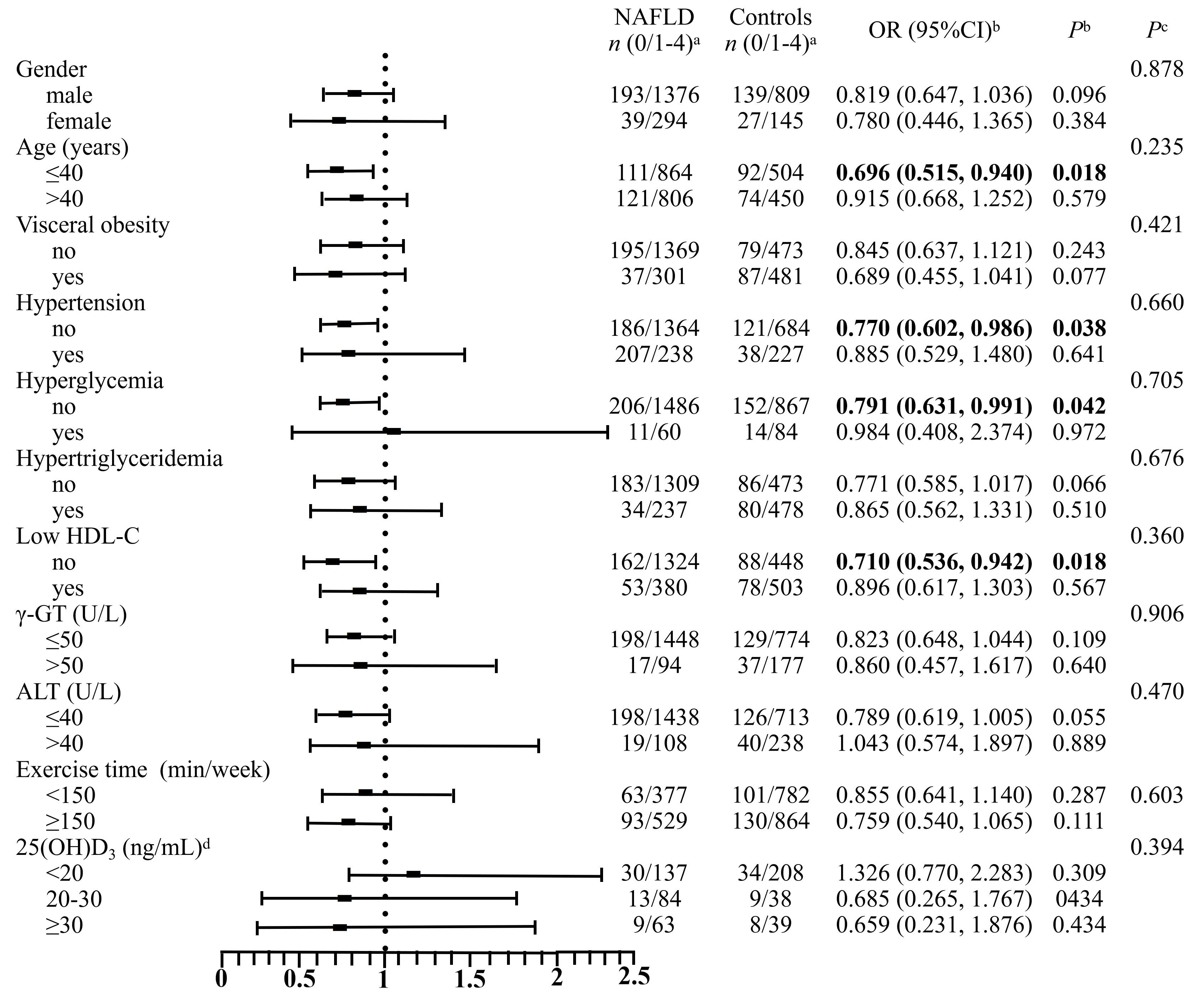


**Supplementary Figure 2. Stratification analyses of associations between combined favorable alleles and NAFLD risk.**

*Abbreviations:* NAFLD, non-alcoholic fatty liver disease; OR, odds ratio; CI, confidence interval; HDL-C, high density liptein cholesterol; γ-GT, γ-glutamyl transpeptidase; ALT, alanine aminotransferase; AST, aspartate aminotransferase; 25(OH)D_3_, 25-hydroxyvitamin D3.

^a^ Favorable allele number.

^b^ Logistic regression analyses with adjustment for gender and age (except the stratification factors of each stratum).

^c^ *P*-value for the heterogeneity test.

^d^ Serum 25(OH)D_3_ levels were measured in 336 NAFLD cases and 336 controls.

Bold type indicates statistically significant results.
